# Prognostic Value of Pre-Treatment CT Radiomics and Clinical Factors for the Overall Survival of Advanced (IIIB–IV) Lung Adenocarcinoma Patients

**DOI:** 10.3389/fonc.2021.628982

**Published:** 2021-05-28

**Authors:** Duo Hong, Lina Zhang, Ke Xu, Xiaoting Wan, Yan Guo

**Affiliations:** ^1^ Department of Radiology, The First Hospital of China Medical University, Shenyang, China; ^2^ GE Healthcare, Beijing, China

**Keywords:** adenocarcinoma of lung, tomography, radiomics, machine learning, overall survival

## Abstract

**Purpose:**

The purpose of this study was to investigate the prognostic value of pre-treatment CT radiomics and clinical factors for the overall survival (OS) of advanced (IIIB–IV) lung adenocarcinoma patients.

**Methods:**

This study involved 165 patients with advanced lung adenocarcinoma. The Lasso–Cox regression model was used for feature selection and radiomics signature building. Then a clinical model was built based on clinical factors; a combined model in the form of nomogram was constructed with both clinical factors and the radiomics signature. Harrell’s concordance index (C-Index) and Receiver operating characteristic (ROC) curves at cut-off time points of 1-, 2-, and 3- year were used to estimate and compare the predictive ability of all three models. Finally, the discriminatory ability and calibration of the nomogram were analyzed.

**Results:**

Thirteen significant features were selected to build the radiomics signature whose C-indexes were 0.746 (95% CI, 0.699 to 0.792) in the training cohort and 0.677 (95% CI, 0.597 to 0.766) in the validation cohort. The C-indexes of combined model achieved 0.799 (95% CI, 0.757 to 0.84) in the training cohort and 0.733 (95% CI, 0.656 to 0.81) in the validation cohort, which outperformed the clinical model and radiomics signature. Moreover, the areas under the curve (AUCs) of the radiomic signature for 2-year prediction was superior to that of the clinical model. The combined model had the best AUCs for 2- and 3-year predictions.

**Conclusions:**

Radiomic signatures and clinical factors have prognostic value for OS in advanced (IIIB–IV) lung adenocarcinoma patients. The optimal model should be selected according to different cut-off time points in clinical application.

## Introduction

Lung cancer, as a leading cause of cancer-related mortality, is responsible for approximately 1.4 million deaths annually throughout the world (1). Non-small cell lung cancer (NSCLC) represents approximately 85% of lung cancers, and adenocarcinoma is the most common histological subtype of NSCLC (2). As NSCLC has no specific early symptoms and signs, 57% of patients present with advanced stage disease at primary diagnosis (3), which may deny patients the opportunity to receive resection and result in a diminished survival time.

Since the 1990s, emergence of chemotherapy with platinum doublets and tyrosine kinase inhibitors (TKIs) has made breakthroughs in the treatment for NSCLC (4); however, the 5-year overall survival (OS) rate is only 5% for those with metastatic disease (5). Thus, the ability to predict clinical outcomes accurately is crucial for clinicians to judge the most appropriate therapies for these patients to improve prognosis. To this end, biomarkers are needed (6).

The tumor node metastasis (TNM) staging system is the most important postoperative prognostic tool that guides treatment, but there are marked variations in responses and prognosis for patients who are undergoing similar treatment in the same stage. The heterogeneity reflects the complexity of the underlying genotype and microenvironment; increasing numbers of -omics studies are being conducted to better understand the complexity (7). Radiomics is an emerging field that converts imaging data into a high-dimensional mineable feature space using a large number of automatically applied algorithms to relate a variety of tumor characteristics (8). Radiomic features are known to pick up the heterogeneity of the tumor (9, 10); since visualization of heterogeneity has been linked to tumor aggressiveness (11), it correlates with poor outcome. Many studies have elucidated the predictive potential of radiomic features for NSCLC prognosis (12). Kirienko et al. identify an images-based radiomic signature capable of predicting disease-free survival (DFS) in NSCLC patients (13); He et al. described a combination of features (size, shape, texture and wavelets) which could predict OS for NSCLC patients (14); but majority of them involved patients of all stages, which might interfere with results because the therapeutic modalities and prognosis between early and advanced-stage patients were of significant difference. Our study limited the subjects to patients with advanced (stage IIIB–IV) lung adenocarcinoma and attempted to predict the OS based on pre-treatment contrast-enhanced computed tomography (CT) radiomics.

## Materials and Methods

### Patients

**Figure 1:**
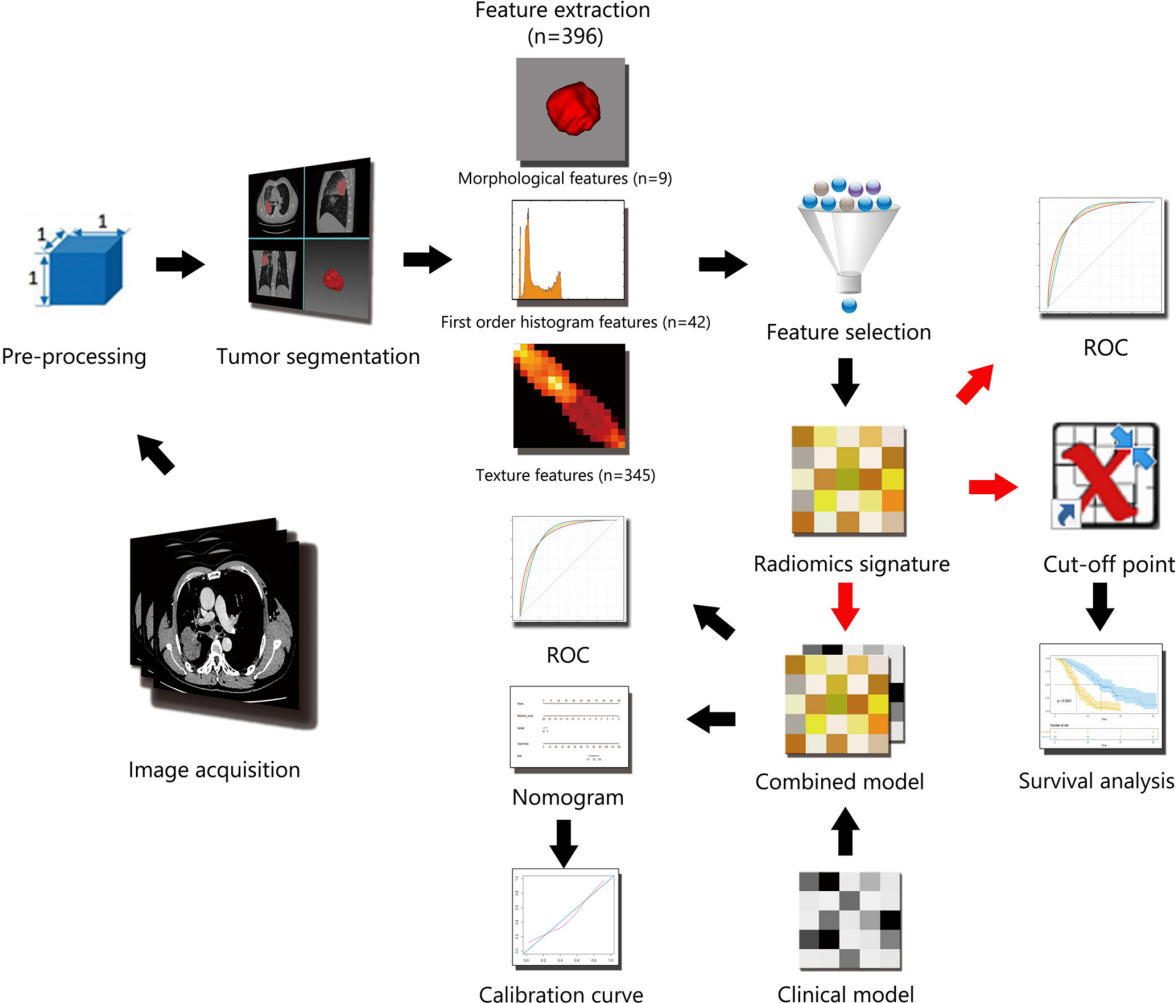
Workflow of the radiomic analysis.

Institutional review board approval was obtained for this retrospective study, with a waiver for the informed consent requirement. A total of 493 consecutive pathologically confirmed advanced stage (IIIB–IV) lung adenocarcinoma patients were recruited retrospectively from January 2014 to December 2017. The inclusion criteria were as follows: (1) age >18; (2) Eastern Cooperative Oncology Group (ECOG) performance status of 0–2; and (3) restricted therapeutic regimens: patients with TKI-sensitive epidermal growth factor receptor (EGFR) mutations or anaplastic lymphoma kinase (ALK) rearrangement accepted TKI therapy initially, and the rest of the patients accepted platinum-based chemotherapy initially. The exclusion criteria were as follows: (1) examination by unassigned CT scanners (n = 127); (2) previous anticancer therapy (n = 25); (3) incomplete clinical data (n = 92); (4) difficulty in distinguishing boundary of regions of interest (ROIs) (n = 51); and (5) loss of follow-up (n = 33). Ultimately, 165 patients were included in this study with no ALK rearrangement patients (**Figure S1**). The clinical data collected for analysis included sex, age, ECOG, TNM stage, smoking status, TKI-sensitive EGFR mutations, tumor diameter, location, margin, lobulation, spiculation, air-bronchogram, pleural invasion, lymph node metastases, brain metastases, liver metastases, and bone metastases. The patients were randomly divided into two individual cohorts for training and validation at a ratio of 7:3 through computer-generated random numbers. The workflow of the radiomic analysis is illustrated in **Figure 1**.

### Image Acquisition

Contrast-enhanced CT images were acquired from Toshiba Aquilion One, Toshiba Aquilion 64 (Toshiba Medical Systems) or Phillips Brilliance iCT 256 (Philips Medical Systems) scanners. The scanning parameters were as follows: 120 kVp; 100–200 mAs; detector collimation of 64, 256, or 320 × 0.625 mm; field of view of 350 × 350 mm; matrix of 512 × 512 and reconstructed slice thickness of 2 mm. Contrast-enhanced CT scanning was performed with a 25-s delay after the injection of 85 ml of non-ionic iodinated contrast material (350 mg iodine/ml, Omnipaque, GE Healthcare). All images were exported to the Picture Archiving and Communication System (PACS) workstation (IMPAX, AGFA).

### Image Pre-Processing

Image pre-processing was performed to enhance feature robustness and reduce feature dependence on scanner variations. Each voxel corresponded to a volume of 1.0 mm * 1.0 mm * 1.0 mm with a linear interpolation algorithm, then a Gaussian filter was used to remove noise. The gray level was consistent across the different scanners; therefore, gray level normalization was not required here.

### Tumor Segmentation and Feature Extraction

Three-dimensional (3D) contours of the tumor regions of interest (ROIs) were delineated manually in reference to pulmonary and mediastinum windows (window width and window level of 1,500 and −450 HU on pulmonary window, while the window width and window level of 400 and 40 HU on mediastinal window). Segmentation was strictly performed by two chest radiologists (W.XT. with 7 years of experience and H.D. with 13 years of experience in chest CT) who were blinded to all patients’ information. The radiologists delineated the boundaries of the tumors on a transversal plane using itk-SNAP (version 3.4.0, www.itk-snap.org) software (**Figure S2**). The image biomarker standardization initiative (IBSI) was regarded as reference and taken into consideration in most of the data processing, images feature, and biomarker selection procedure.

A total of 396 radiomic features were generated automatically using in-house software (Artificial Intelligence Kit, A.K., GE Healthcare) from ROIs. Features were classified into the following three categories: (a) morphological features (n = 9); (b) first-order features (intensity features, n = 42); and (c) texture features (n = 345). The details are given in **Figure S3**.

Inter-/intra-class correlation coefficients (ICCs) were used to evaluate the inter-observer and intra-observer agreement. To assess inter-observer reproducibility, the ROIs of 30 randomly chosen images were performed by the two chest radiologists independently; to evaluate intra-observer reproducibility, they repeated the same procedure at an one-month interval. An ICC >0.75 was considered as good agreement. Stable and reproducible features were entered in the subsequent analysis.

### Feature Selection and Radiomic Signature Building

Least absolute shrinkage and selection operator (LASSO) Cox regression analysis was utilized to select effective and predictable features and establish a model in the training cohort. Features with non-zero coefficients were chosen based on 10-fold cross-validation (**Figure S4**). The radiomics signature (Rad-score), which was calculated *via* a linear combination of the selected features that had been weighted by their respective coefficients, represented quantitative ROI characteristics of each patient.

### Validation of the Radiomic Signature

(1) The patients were divided into high- and low-risk subgroups in the training and validation cohorts according to the Rad-score, and the optimized threshold values were determined using X-Tile software (version 3.6.1, Yale University). Then, Kaplan–Meier OS curves and log-rank analyses were performed to assess the prognosis of subgroups. (2) The receiver operating characteristic (ROC) curves were plotted, and areas under the curves (AUCs) were calculated for predictive validity assessment of survival at 1-, 2- and 3-year time points in the training and validation cohorts. (3) The validation cohort was further divided into mutated EGFR subgroup and wild type EGFR subgroup. Harrell’s concordance index (C-index) and ROC curves for 1-, 2- and 3-year survival were used to compare the performances of radiomic model in both subgroups.

### Clinical Model Building and Validation

The clinical model was built by Cox proportional hazard regression to compare with the radiomic signature. Sex, age, ECOG, TNM stage, smoking status, TKI-sensitive EGFR mutations, tumor diameter, location, margin, lobulation, spiculation, air-bronchogram, pleural invasion, lymph node metastases, brain metastases, liver metastases, and bone metastases in the training cohort were first analyzed by univariate Cox regression. Only significant factors (p < 0.05) from univariate Cox regression were entered into the multivariate Cox regression analysis. AUCs were calculated for the clinical model in the same way and compared with that of radiomic signature using DeLong test.

### Combined Model Construction and Validation

The combined model in the form of nomogram for 1-, 2- and 3-year overall survival rate predictions was generated on the basis of the Rad-score and the clinical factors with P <0.05 in univariate Cox regression. Backward multivariate cox regression was used again, and the factors with P <0.05 were incorporated into the nomogram. The discriminative power of the predictive model was evaluated by C-index with 95% confidence intervals and AUCs in both cohorts. The calibration curves were plotted to explore the calibration degree of the combined model for the 1-, 2- and 3-year OS rates.

### Statistical Analysis

All statistical analyses were performed using R statistical software version 3.6.2. The “glmnet” package was used for executing the LASSO Cox algorithm. For the baseline characteristic analyses, the normality of data was assessed by the Shapiro–Wilk test. Differences between the training and validation cohorts were assessed by using independent-sample *t*-test and chi-square test, where appropriate. Performances of the models were evaluated by C-index. A two-sided *p*-value <0.05 was considered statistically significant for all comparisons.

## Results

### Patients

A total of 165 patients were enrolled in the study. The cohort consisted of 94 men and 71 women with a mean age of 58.1 years (range of 34–78 years). The longest follow-up period was 72 months, and the mean was 19.2 months. The training cohort included 115 patients; the validation cohort included 50 patients. Patients’ clinical characteristics are reported in **Table 1**. There were no significant differences except ECOG performance status in clinical factors between the two cohorts.

**Table 1:** Demographic data of patients in the training and validation cohorts.

	Training cohort (N = 115)	Validation cohort (N = 50)	*p*
Sex/No. (%)			0.611†
Male	67(58.3)	27(54)	
Female	48(41.7)	23(46)	
Age/Mean ± SD	57.3 ± 9.6	60 ± 8.7	0.085‡
ECOG/No. (%)			0.006†*
0-1	75(65.2)	43(86)	
2	40(34.8)	7(14)	
Smoking status/No. (%)			0.278†
Smoker	36(31.3)	20(40)	
Never	79(68.7)	30(60)	
Stage/No. (%)			0.968†
IIIB	21(18.3)	9(18)	
IV	94(81.7)	41(82)	
Treatment method /No. (%)			0.797†
TKIs	55(47.8)	25(50)	
Chemotherapy	60(52.2)	25(50)	
Tumor diameter (cm)			0.902†
<5	54(47)	24(48)	
≥5	61(53)	26(52)	
Location/No. (%)			0.81†
Central	46(40)	21(42)	
Peripheral	69(60)	29(58)	
Margin/No. (%)			0.061†
well-defined	99(86)	48(96)	
ill-defined	16(14)	2(4)	
Lobulation/No. (%)			0.324†
Yes	97(84)	39(78)	
No	18(16)	11(22)	
Spiculation/No. (%)			0.755†
Yes	49(42.6)	20(40)	
No	66(57.4)	30(60)	
Air-bronchogram/No. (%)			0.916†
Yes	29(25.2)	13(26)	
No	86(74.8)	37(74)	
Pleural invasion/No. (%)			0.447†
Yes	28(24.3)	15(30)	
No	87(75.6)	35(70)	
Lymph node metastasis /No. (%)			0.809†
Yes	69(60)	31(62)	
No	46(40)	19(38)	
Brain metastases /No. (%)			0.488†
Yes	16(13.9)	5(10)	
No	99(86.1)	45(90)	
Liver metastases /No. (%)			0.602†
Yes	17(14.8)	9(18)	
No	98(85.2)	41(82)	
Bone metastases /No. (%)			0.933†
Yes	19(16.5)	8(16)	
No	96(83.5)	42(84)	
Rad-score/Mean ± SD	−0.118 ± 0.653	0.177 ± 0.656	0.598‡
High-low risk/No. (%)			0.966†
High risk	41(35.7)	18(36)	
Low risk	74(64.3)	32(64)	

† Chi-square test was used.

‡ Independent-samples t-test was used.

*p < 0.05 was considered statistically significant.

### Feature Selection and Radiomic Signature Building

After assessing the reproducibility, 324 features with both inter- and intra-class correlation coefficients >0.75 were reserved. In the training cohort, 13 features were evaluated to construct a radiomic signature through the LASSO Cox algorithm: [1] Range; [2] skewness; [3] GLCMEntropy_ AllDirection_offset1_SD; [4] GLCMEntropy_angle135_offset1; [5] Correlation_AllDirection_offset4_SD; [6] GLCMEnergy_angle45_offset7; [7] GLCMEntropy_angle45_offset7; [8] sumAverage; [9] ShortRunLowGreyLevelEmphasis_AllDirection_offset1_SD; [10] ShortRunEmphasis-_AllDirection_offset4_SD; [11] ShortRunHighGreyLevelEmphasis_AllDirection_offset7_SD; [12] ShortRunHighGreyLevelEmphasis_angle0_offset7; and [13] Sphericity. The formula of Rad-score is illustrated in **Table S1**.

### Validation of the Radiomic Signature

The C-indexes of the radiomic signature were 0.746 (95% CI, 0.699 to 0.792) in the training cohort and 0.677 (95% CI, 0.597 to 0.766) in the validation cohort.

**Figure 2:**
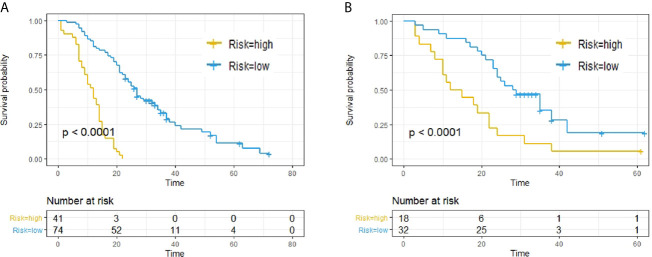
Predictive capacity of radiomic signatures. Kaplan–Meier curves showed that radiomics signatures could effectively discriminate patients with low risk from those with high risk. **(A)** Training cohort. **(B)** Validation cohort.

**Figure 3:**
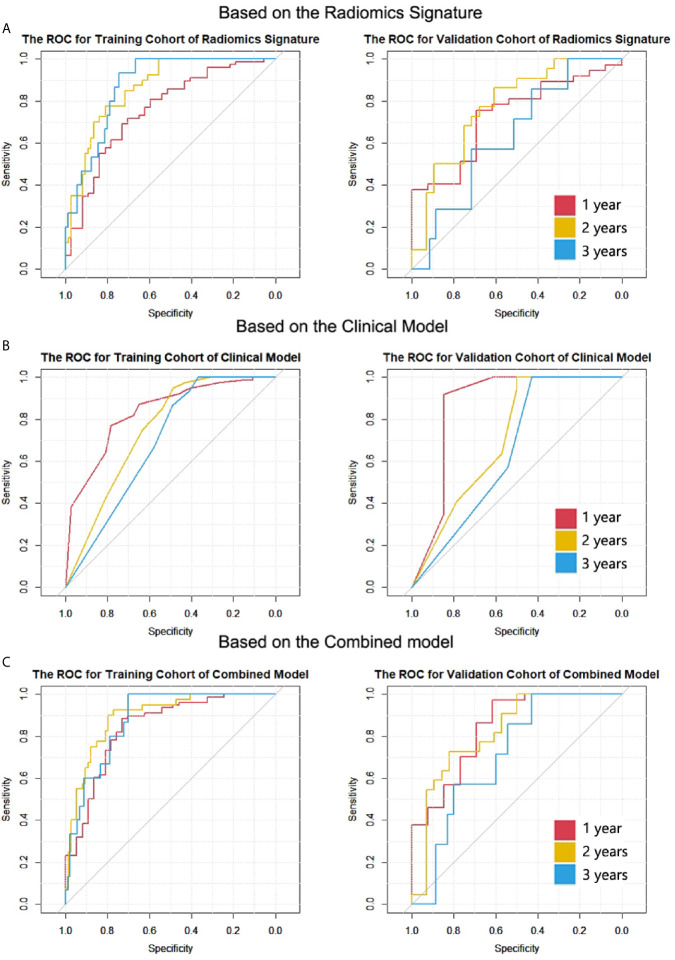
The ROC curves of the two cohorts for 1-, 2- and 3-year survival in all models. **(A)** Radiomic signature. **(B)** Clinical model. **(C)** Combined model. The numbers of patients who died after 1-, 2- and 3-year cut-off time were 78, 40 and 15 in the training cohort; the numbers were 37, 22, and 7 in the validation cohort.

(1) The patients were classified into high- and low-risk subgroups according to the Rad-score at a cut-off point of 0.15 according to X-Tile. Kaplan–Meier analysis revealed significantly different subgroup OS in both training cohort (p < 0.0001, log-rank test) and validation cohort (p < 0.0001, log-rank test), as shown in **Figure 2**.

(2) The ROC curves of the two cohorts for 1-, 2- and 3-year survival are plotted in **Figure 3A**.

(3) The C-index for mutated EGFR subgroup was 0.629 (95% CI, 0.476 to 0.782) in validation cohort; the C-index for wild type EGFR subgroup was 0.662 (95% CI, 0.537 to 0.787) in validation cohort. The AUCs of the subgroups for 1-, 2- and 3-year survival are shown in **Table S2**. Although there was no significant difference by the DeLong test in all pairs, the AUCs of wild type EGFR subgroup were superior to that of mutated EGFR subgroup for all cut-off time points.

### Clinical Model Building and Validation

The variables with p values <0.05 in the univariate analysis, namely, ECOG, TKI-sensitive EGFR mutations (treatment methods), pleural invasion, and brain metastases, were entered into the multivariate analysis. The multivariate Cox proportional hazard model showed that all the entered factors were identified as independent predictors of OS (**Table 2**).

**Table 2:** Univariate and multivariate analyses for clinical data.

Risk factor	Univariate			Multivariate		
	HR	95% CI	*p*	HR	95% CI	*p*
Sex (M/F)	1.355	0.907–2.026	0.138			
Age (>60y/≤60y)	0.917	0.609–1.379	0.678			
ECOG (0–1/2)	0.299	0.196–0.456	<0.01*	0.358	0.222–0.576	<0.01*
Smoking status (Yes/No)	1.195	0.783–1.823	0.409			
Stage (IIIB/IV)	0.638	0.372–1.097	0.104			
Tumor diameter (≥5 cm/<5 cm)	1.074	0.723–1.596	0.722			
Location (Central/Peripheral)	1.245	0.834–1.859	0.284			
Margin (well-defined/ill-defined)	0.873	0.493–1.547	0.643			
Lobulation (Yes/No)	1.285	0.715–2.307	0.401			
Spiculation (Yes/No)	1.394	0.931–2.087	0.106			
Air-bronchogram (Yes/No)	1.32	0.849–2.053	0.218			
Pleural invasion (Yes/No)	1.589	1.017–2.483	0.042*	1.888	1.196–2.979	<0.01*
Lymph node metastasis (Yes/No)	1.334	0.888–2.002	0.165			
Brain metastases (Yes/No)	5.236	2.924–9.376	<0.01*	3.417	1.798–6.493	<0.01*
Liver metastases (Yes/No)	1.579	0.921–2.708	0.097			
Bone metastases (Yes/No)	1.482	0.864–2.544	0.153			
Treatment methods (TKIs/Chemotherapy)	0.649	0.437–0.965	0.033*	0.533	0.354–0.803	<0.01*

*p < 0.05 was considered statistically significant.

**Figure 4:**
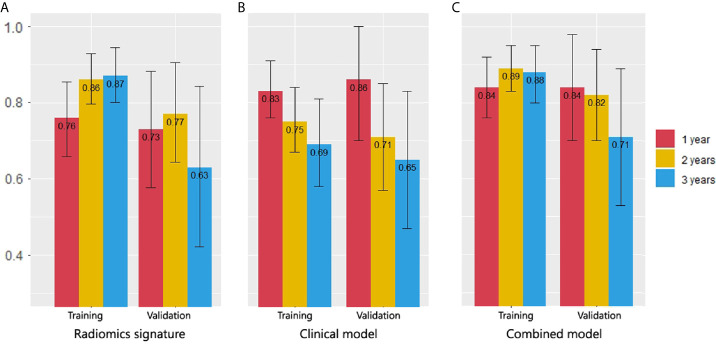
Comparison of 1-, 2- and 3-year survival AUCs in all models. **(A)** Radiomic signature. **(B)** Clinical model. **(C)** Combined model. In the validation cohort, the AUC of the radiomics signature for 1-year prediction was inferior to that of the clinical model, but the 2-year prediction was superior to that of the clinical model. The combined model had the best AUCs in 2- and 3-year predictions.

For the clinical model, the C-indexes were 0.718 (95% CI, 0.669 to 0.766) in the training cohort and 0.698 (95% CI, 0.603 to 0.792) in the validation cohort. The ROC curves for the clinical model are depicted in **Figure 3B**. The comparison of AUCs for radiomic signatures and the clinical model are shown in **Figure 4**. In the validation cohort, although there was no significant difference by the DeLong test in all pairs, the AUC of the radiomic signature for 1-year prediction was inferior to that of the clinical model, but the 2-year prediction was superior to that of the clinical model. The prediction efficiencies of both models for 3-year survival were not satisfactory.

### Combined Model Construction and Validation

**Figure 5:**
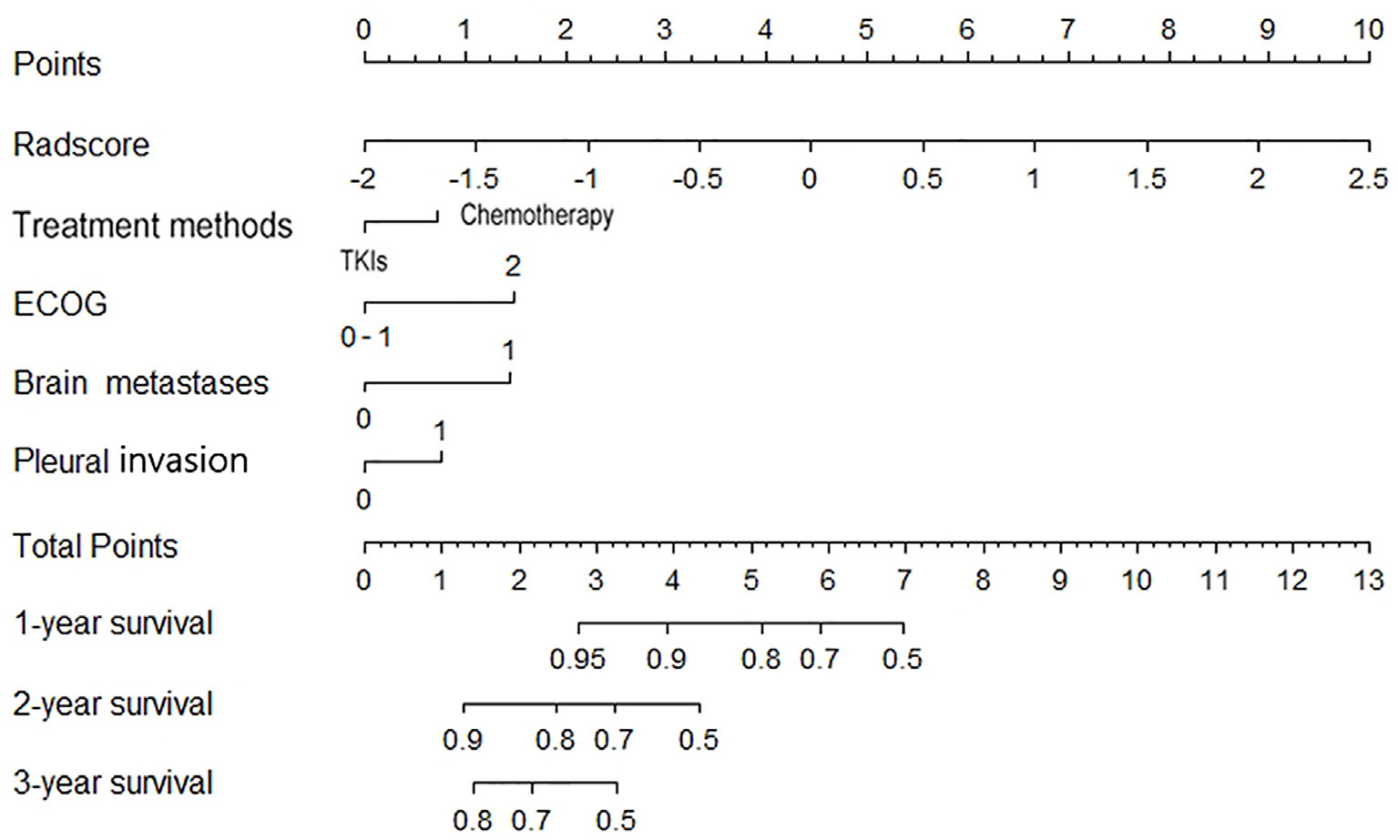
Nomogram for estimating the 1-, 2- and 3-year survival rates.

Clinical parameters with p <0.05 in the univariate Cox regression (ECOG, treatment methods pleural invasion and brain metastases) and Rad-score were included in the construction of the combined model using backward multivariate Cox regression (**Table 3**). The nomogram was showed in **Figure 5**.

**Table 3:** Results of multivariate Cox regression for combined model.

	Coefficient	HR	95% CI		*p*
			Lower	Upper	
Rad-score	1.499	4.475	2.899	6.919	<0.01*
ECOG (0–1/2)	-0.921	0.398	0.245	0.646	<0.01*
Pleural invasion (Yes/No)	0.475	1.608	1.018	2.54	0.042*
Brain metastases (Yes/No)	0.899	2.458	1.267	4.769	<0.01*
Treatment methods (TKIs/Chemotherapy)	-0.445	0.641	0.424	0.968	0.034*

*p < 0.05 was considered statistically significant.

**Figure 6:**
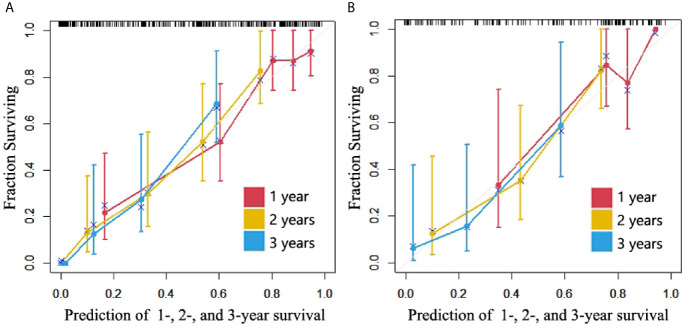
Calibration curves of the nomogram. **(A)** Training cohort. **(B)** Validation cohort.

The C-indexes of the combined model were 0.799 (95% CI, 0.757 to 0.84) in the training cohort and 0.733 (95% CI, 0.656 to 0.81) in the validation cohort, which were higher than those of the other two models. ROC curves are delineated in **Figure 3C**. **Figure 4C** shows that the combined model improved the accuracy for 2- and 3-year survival predictions compared with the radiomics and clinical models. The calibration curves of the nomogram demonstrated good consistency between predicted and observed results (**Figure 6**).

## Discussion

The present study explored whether a radiomic approach could be used to generate prognostic biomarkers of OS for advanced lung adenocarcinoma patients. Three models (radiomic signature, clinical model, and combined model) were constructed and compared. We found that the radiomic signature and clinical model had similar predictive performance in the validation cohort (C-index, 0.677 and 0.698), and they were mutually complementary for predicting 1- and 2-year survival. The combined model provided a better and balanced estimation (C-index, 0.733) in the validation cohort.

Many studies have attempted to utilize different features to “phenotype” tumor and predict the outcomes of patients with lung cancer (10, 15). Huang et al. found a correlation between radiomics biomarkers on CT and disease-free survival (DFS) in early stage (I or II) NSCLC and the C-index of the model was 0.72 (95% CI, 0.71 to 0.73) (16). Yang et al. developed a radiomic nomogram based on the 2D and 3D CT features which yielded a C-index of 0.731 (95% CI, 0.626 to 0.836) to predict the survival of NSCLC patients (17). Our study tried to construct a model to predict OS for patients with advanced (IIIB–IV) lung adenocarcinoma (C-index of combined model, 0.733, 95%CI, 0.656 to 0.81), who starved for a more accurate prediction to improve initial therapeutic regimens. Furthermore, we compared the accuracy of the radiomic model, clinical model and combined model in the prediction for 1-, 2- and 3-year survival and draw a conclusion that the optimal model should be selected according to the cut-off time points.

In our research, the clinical model had the peak accuracy in the 1-year prediction (AUC = 0.864, 95% CI, 0.697 to 1) because death from the existence of brain metastases (HR = 5.236, 95% CI, 2.924 to 9.376) and higher ECOG score (HR = 3.344, 95% CI, 2.193 to5.102) could occur quickly. The median OS for brain metastasis patients was only 8 months; the median OS for ECOG = 2 patients was only 9 months; however, 1 year later, when the impact of these factors weakened, the prediction accuracy of the clinical model fell sharply (AUC = 0.712, 95% CI, 0.573 to 0.853). The radiomic signature had complementary advantages in the 2-year prediction with clinical model (AUC = 0.774, 95% CI = 0.644 to 0.901); the combined model exhibited the best AUC for 2-year prediction (AUC = 0.82, 95% CI, 0.701 to 0.939).

There are different treatment methods in mutated EGFR group and wild type EGFR group (18), so generally, their prognosis was studied separately. However, many previous studies have shown that radiomic has a high accuracy in distinguishing wild and mutated EGFR NSCLC patients (19, 20), therefore, we combined two groups to increase the universality of the models. We also performed subgroup analysis which indicated that the radiomic signature own higher discrimination capacity for wild type EGFR group than mutated EGFR subgroup for all cut-off time points.

In contrast to findings in previous articles, TNM stage was not an independent risk factor in the clinical model in our study because the inclusion criteria were limited to stage IIIB and IV patients, whose OS times were not significantly different (median OS, 24 *vs* 19 months, p = 0.38).

Some limitations of this study have to be acknowledged. First, it was a retrospective study with a relatively small number of samples, which might cause instability in feature values (21); second, histologic grade and subtype were recognized prognostic factors (13, 22–24), but they were not tested in our study due to the unavailability of whole tumor specimens through transthoracic or transbronchoscopic biopsy (25); third, anti-PD-1/L1 monotherapy has already been approved by the US Food and Drug Administration (FDA) for treatment of patients with advanced lung adenocarcinoma (26), but it was not considered in our study. Future study with larger samples and anti-PD-1/L1 monotherapy is warranted.

## Conclusion

The radiomic signatures and clinical factors have prognostic value for OS in advanced (IIIB–IV) lung adenocarcinoma patients. The results of the radiomic signature and the clinical model in predicting 1- and 2-year survival were complementary and the optimal model should be selected according to the cut-off time.

## Data Availability Statement

The raw data supporting the conclusions of this article will be made available by the authors without undue reservation.

## Ethics Statement

The studies involving human participants were reviewed and approved by the Ethics Committee of The First Hospital of China Medical University. Written informed consent for participation was not required for this study in accordance with the national legislation and the institutional requirements.

## Author Contributions

DH conceived of the project, performed the experiments, and wrote the paper. YG and XW analyzed the data. LZ and KX provided expert guidance and reviewed the manuscript. All authors contributed to the article and approved the submitted version.

## Conflict of Interest

YG was employed by the company GE Healthcare, China.

The remaining authors declare that the research was conducted in the absence of any commercial or financial relationships that could be construed as a potential conflict of interest.

## Supplementary Material

Supplementary Figure 1 Flowchart of criteria for patient inclusion.

Supplementary Figure 2 The demonstration of our radiologists working on the tumor segmentation with itk-SNAP.

Supplementary Figure 3 Details for three types of radiomic features extracted from CT images.

Supplementary Figure 4 Feature selection using LASSO regression model in the training dataset.

## References

[1] She J Yang P Hong Q Bai C. Lung Cancer in China: Challenges and Interventions. Chest (2013) 143(4):1117–26. 10.1378/chest.11-2948 23546484

[2] Hong D Xu K Zhang L Wan X Guo Y. Radiomics Signature as a Predictive Factor for EGFR Mutations in Advanced Lung Adenocarcinoma. Front Oncol (2020) 10:28. 10.3389/fonc.2020.00028 32082997 PMC7005234

[3] Siegel RL Miller KD Jemal A. Cancer Statistics, 2020. CA Cancer J Clin (2020) 70(1):7–30. 10.3322/caac.21590 31912902

[4] Yoshida T Kuroda H Oya Y Shimizu J Horio Y Sakao Y. Clinical Outcomes of Platinum-Based Chemotherapy According to T790M Mutation Status in EGFR-Positive Non-Small Cell Lung Cancer Patients After Initial EGFR-TKI Failure. Lung Cancer (2017) 109:89–91. 10.1016/j.lungcan.2017.05.001 28577956

[5] Singh R Peng S Viswanath P Sambandam V Shen L Rao X. Non-Canonical cMet Regulation by Vimentin Mediates Plk1 Inhibitor-Induced Apoptosis. EMBO Mol Med (2019) 11(5):e9960. 10.15252/emmm.201809960 31040125 PMC6505578

[6] Van Timmeren JE Rth L Van EW Reymen B Oberije C Monshouwer R. Survival Prediction of Non-Small Cell Lung Cancer Patients Using Radiomics Analyses of Cone-Beam CT Images. Radiother Oncol (2017) 123(3):363. 10.1016/j.radonc.2017.04.016 28506693

[7] Ming X Oei RW Zhai R Kong F Du C Hu C. MRI-Based Radiomics Signature Is a Quantitative Prognostic Biomarker for Nasopharyngeal Carcinoma. Sci Rep (2019) 9(1):10412. 10.1038/s41598-019-46985-0 31320729 PMC6639299

[8] Wu Y Xu L Yang P Lin N Huang X Pan W. Survival Prediction in High-grade Osteosarcoma Using Radiomics of Diagnostic Computed Tomography. EBioMedicine (2018) 34:27–34. 10.1016/j.ebiom.2018.07.006 30026116 PMC6116348

[9] Wei W Wang K Liu Z Tian K Wang L Du J. Radiomic Signature: A Novel Magnetic Resonance Imaging-Based Prognostic Biomarker in Patients With Skull Base Chordoma. Radiother Oncol (2019) 141:239–46. 10.1016/j.radonc.2019.10.002 31668985

[10] Thawani R Mclane M Beig N Ghose S Prasanna P Velcheti V. Radiomics and Radiogenomics in Lung Cancer: A Review for the Clinician. Lung Cancer (2018) 115:34. 10.1016/j.lungcan.2017.10.015 29290259

[11] Wei W Liu Z Rong Y Zhou B Bai Y Wei W. A Computed Tomography-Based Radiomic Prognostic Marker of Advanced High-Grade Serous Ovarian Cancer Recurrence: A Multicenter Study. Front Oncol (2019) 9:255. 10.3389/fonc.2019.00255 31024855 PMC6465630

[12] Zhang Y Oikonomou A Wong A Haider MA Khalvati F. Radiomics-Based Prognosis Analysis for Non-Small Cell Lung Cancer. Sci Rep (2017) 7:46349. 10.1038/srep46349 28418006 PMC5394465

[13] Kirienko M Cozzi L Antunovic L Lozza L Fogliata A Voulaz E. Prediction of Disease-Free Survival by the PET/CT Radiomic Signature in Non-Small Cell Lung Cancer Patients Undergoing Surgery. Eur J Nucl Med Mol Imag (2018) 45(2):207–17. 10.1007/s00259-017-3837-7 28944403

[14] He B Zhao W Pi JY Han D Jiang YM Zhang ZG. A Biomarker Basing on Radiomics for the Prediction of Overall Survival in Non–Small Cell Lung Cancer Patients. Respir Res (2018) 19:199. 10.1186/s12931-018-0887-8 30305102 PMC6180390

[15] Wang L Dong T Xin B Xu C Guo M Zhang H. Integrative Nomogram of CT Imaging, Clinical, and Hematological Features for Survival Prediction of Patients With Locally Advanced Non-Small Cell Lung Cancer. Eur Radiol (2019) 29(6):2958–67. 10.1007/s00330-018-5949-2 30643940

[16] Huang Y Liu Z He L Chen X Pan D Ma Z. Radiomics Signature: A Potential Biomarker for the Prediction of Disease-Free Survival in Early-Stage (I or II) Non-Small Cell Lung Cancer. Radiology (2016) 281(3):947–57. 10.1148/radiol.2016152234 27347764

[17] Yang L Yang J Zhou X Huang L Zhao W Wang T. Development of a Radiomics Nomogram Based on the 2D and 3D CT Features to Predict the Survival of Non-Small Cell Lung Cancer Patients. Eur Radiol (2019) 29(5):2196–206. 10.1007/s00330-018-5770-y 30523451

[18] Mei D Luo Y Wang Y Gong J. CT Texture Analysis of Lung Adenocarcinoma: Can Radiomic Features be Surrogate Biomarkers for EGFR Mutation Statuses. Cancer Imag (2018) 18(1):52. 10.1186/s40644-018-0184-2 PMC6295009 30547844

[19] Liu G Xu Z Ge Y Jiang B Groen H Vliegenthart R. 3D Radiomics Predicts EGFR Mutation, Exon-19 Deletion and Exon-21 L858R Mutation in Lung Adenocarcinoma. Transl Lung Cancer Res (2020) 9(4):1212–24. 10.21037/tlcr-20-122 PMC7481623 32953499

[20] Lu X Li M Zhang H Hua S Meng F Yang H. A Novel Radiomic Nomogram for Predicting Epidermal Growth Factor Receptor Mutation in Peripheral Lung Adenocarcinoma. Phys Med Biol (2020) 65(5):055012. 10.1088/1361-6560/ab6f98 31978901

[21] Kim J Choi SJ Lee SH Lee HY Park H. Predicting Survival Using Pretreatment CT for Patients With Hepatocellular Carcinoma Treated With Transarterial Chemoembolization: Comparison of Models Using Radiomics. AJR Am J Roentgenol (2018) 211(5):1026–34. 10.2214/AJR.18.19507 30240304

[22] Yang M She Y Deng J Wang T Ren Y Su H. CT-Based Radiomics Signature for the Stratification of N2 Disease Risk in Clinical Stage I Lung Adenocarcinoma. Transl Lung Cancer Res (2019) 8(6):876–85. 10.21037/tlcr.2019.11.18 PMC6976374 32010566

[23] Yasukawa M Sawabata N Kawaguchi T Kawai N Nakai T Ohbayashi C. Histological Grade: Analysis of Prognosis of Non-Small Cell Lung Cancer After Complete Resection. In Vivo (2018) 32(6):1505–12. 10.21873/invivo.11407 PMC6365755 30348709

[24] Tsao MS Marguet S Le Teuff G Lantuejoul S Shepherd FA Seymour L. Subtype Classification of Lung Adenocarcinoma Predicts Benefit From Adjuvant Chemotherapy in Patients Undergoing Complete Resection. J Clin Oncol (2015) 33(30):3439–46. 10.1200/jco.2014.58.8335 PMC4606061 25918286

[25] Park S Lee SM Noh HN Hwang HJ Kim S Do KH. Differentiation of Predominant Subtypes of Lung Adenocarcinoma Using a Quantitative Radiomics Approach on CT. Eur Radiol (2020) 30:4883–92. 10.1007/s00330-020-06805-w 32300970

[26] Huang D Cui P Huang Z Wu Z Tao H Zhang S. Anti-PD-1/L1 Plus Anti-Angiogenesis Therapy as Second-Line or Later Treatment in Advanced Lung Adenocarcinoma. J Cancer Res Clin Oncol (2021) 147(3):881–91. 10.1007/s00432-020-03380-x PMC11801922 32909095

