# Supplementary figures and images for: Prognostic Value of Pre-Treatment CT Radiomics and Clinical Factors for the Overall Survival of Advanced (IIIB–IV) Lung Adenocarcinoma Patients
Source: Front Oncol. 2021 May 28;11:628982. doi: 10.3389/fonc.2021.628982 (PMC8193844; doi:10.3389/fonc.2021.628982)

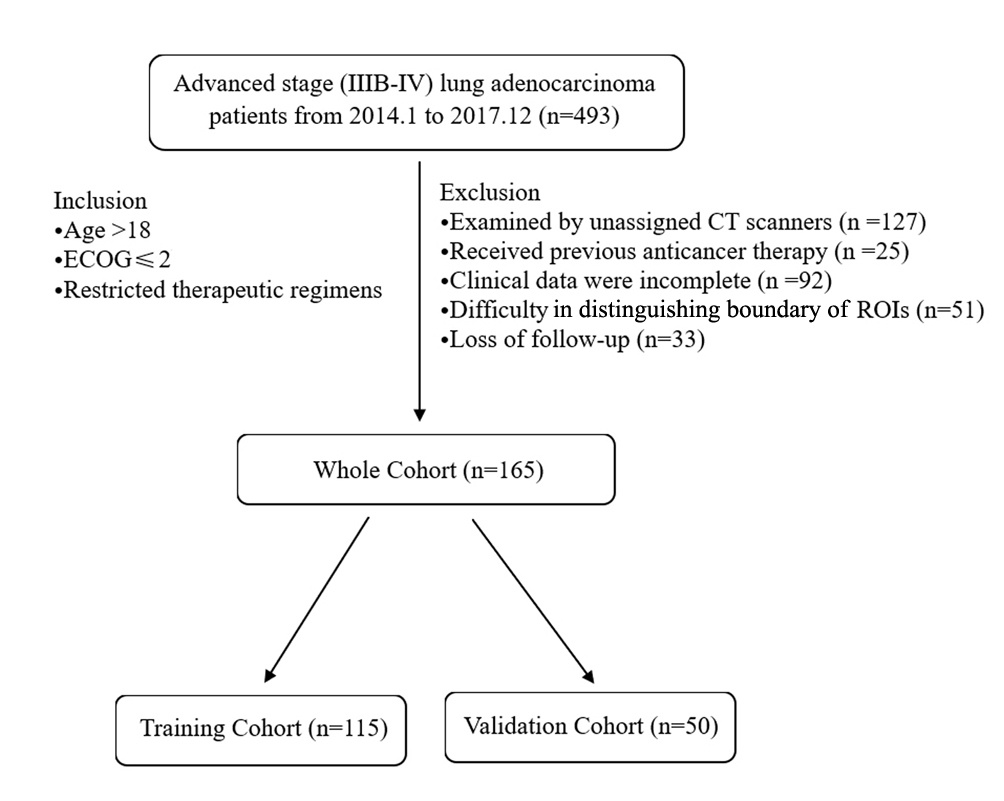

Supplement: Supplementary Figure 1 — Flowchart of criteria for patient inclusion. [file Image_1.jpeg]

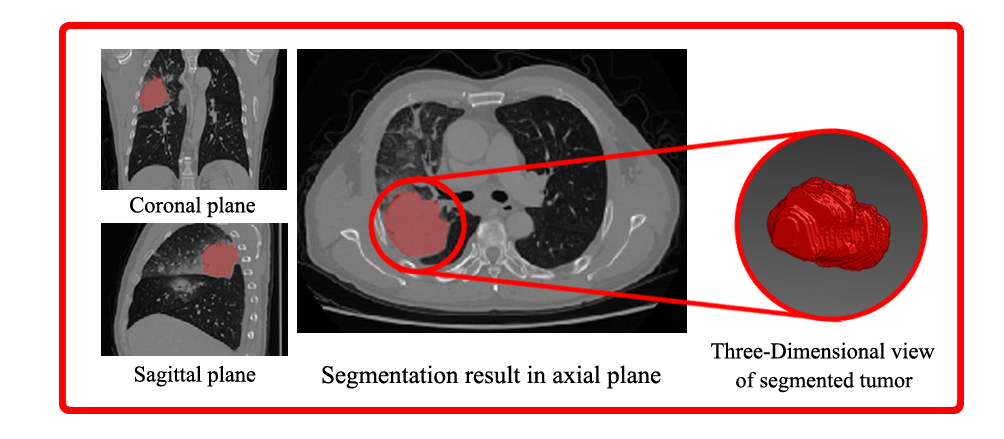

Supplement: Supplementary Figure 2 — The demonstration of our radiologists working on the tumor segmentation with itk-SNAP. [file Image_2.jpeg]

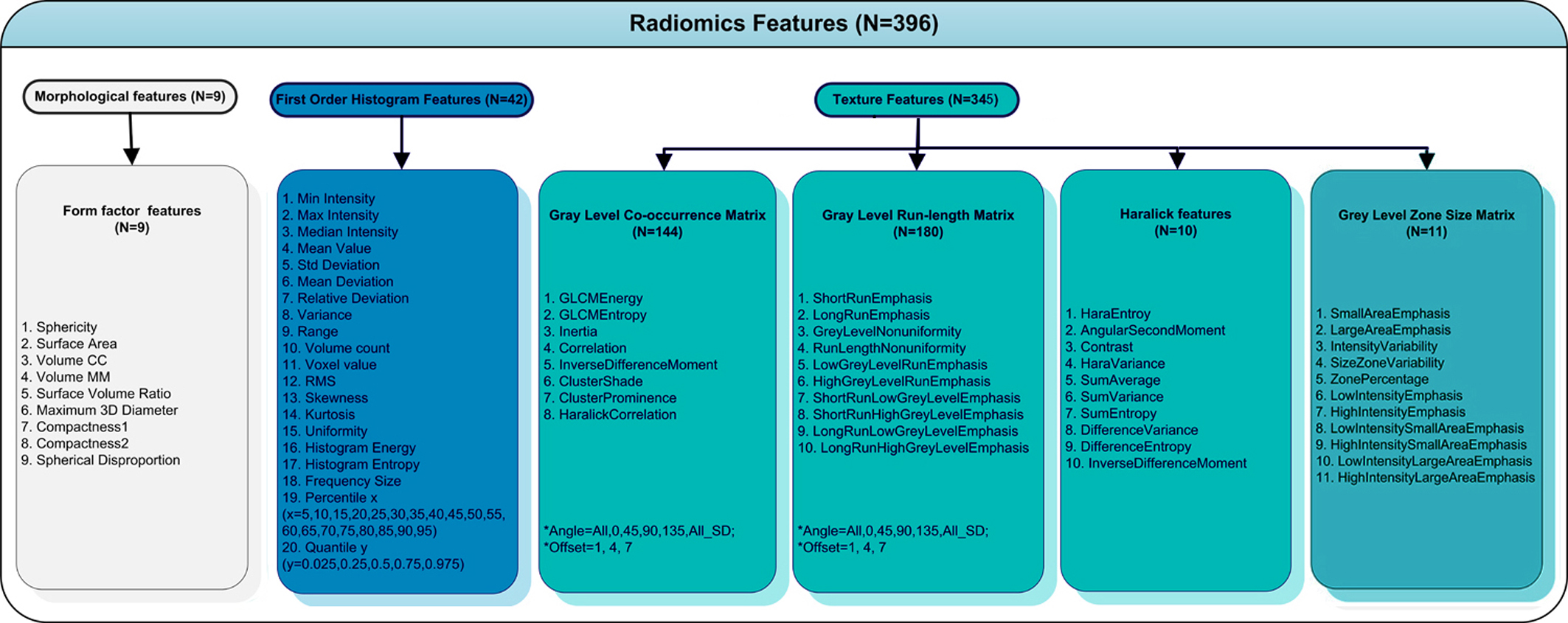

Supplement: Supplementary Figure 3 — Details for three types of radiomic features extracted from CT images. [file Image_3.jpeg]

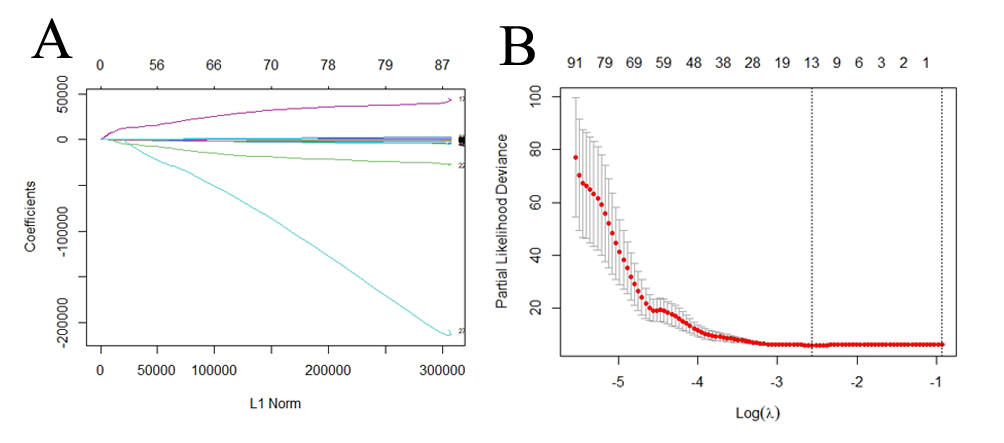

Supplement: Supplementary Figure 4 — Feature selection using LASSO regression model in the training dataset. [file Image_4.jpeg]
