# Supplementary material for: Prognostic Value of Pre-Treatment CT Radiomics and Clinical Factors for the Overall Survival of Advanced (IIIB–IV) Lung Adenocarcinoma Patients
Source: Front Oncol. 2021 May 28;11:628982. doi: 10.3389/fonc.2021.628982 (PMC8193844; doi:10.3389/fonc.2021.628982)
Supplement: Supplementary file 5 [file Table_1.docx]

**Table S1**┃Selected features and their coefficients

|  | Coefficients | Features |
| --- | --- | --- |
| 1 | -1.040024e-03 | Range |
| 2 | 4.562813e-02 | skewness |
| 3 | -2.580377e-01 | GLCMEntropy_AllDirection_offset1_SD |
| 4 | -1.494402e-01 | GLCMEntropy_angle135_offset1 |
| 5 | 2.366126e+02 | Correlation_AllDirection_offset4_SD |
| 6 | 1.02955 | GLCMEnergy_angle45_offset7 |
| 7 | -6.037505e-02 | GLCMEntropy_angle45_offset7 |
| 8 | -3.953274e-01 | sumAverage |
| 9 | 1.772245e+03 | ShortRunLowGreyLevelEmphasis_AllDirection_offset1_SD |
| 10 | 1.322400e+02 | ShortRunEmphasis_AllDirection_offset4_SD |
| 11 | -2.185730e-04 | ShortRunHighGreyLevelEmphasis_AllDirection_offset7_SD |
| 12 | -5.886887e-07 | ShortRunHighGreyLevelEmphasis_angle0_offset7 |
| 13 | 3.462768 | Sphericity |
