# Supplementary material for: Prognostic Value of Pre-Treatment CT Radiomics and Clinical Factors for the Overall Survival of Advanced (IIIB–IV) Lung Adenocarcinoma Patients
Source: Front Oncol. 2021 May 28;11:628982. doi: 10.3389/fonc.2021.628982 (PMC8193844; doi:10.3389/fonc.2021.628982)
Supplement: Supplementary file 6 [file Table_2.docx]

**TABLE┃ S2** The AUCs of the subgroups for 1-, 2- and 3-year survival

|  | Mutated EGFR subgroup  AUC (95% CI) | Wild type EGFR subgroup  AUC (95% CI) | *p*† |
| --- | --- | --- | --- |
| 1-year | 0.717 (0.451-0.984) | 0.728 (0.509-0.932) | 0.985 |
| 2-year | 0.684 (0.413-0.955) | 0.870 (0.705-1.000) | 0.257 |
| 3-year | 0.539 (0.234-0.843) | 0.795 (0.381-1.000) | 0.333 |
